# RhoA of the Rho Family Small GTPases Is Essential for B Lymphocyte Development

**DOI:** 10.1371/journal.pone.0033773

**Published:** 2012-03-16

**Authors:** Shuangmin Zhang, Xuan Zhou, Richard A. Lang, Fukun Guo

**Affiliations:** 1 Division of Experimental Hematology and Cancer Biology, Children's Hospital Research Foundation, Cincinnati, Ohio, United States of America; 2 Division of Pediatric Ophthalmology, Children's Hospital Research Foundation, Cincinnati, Ohio, United States of America; University of Alabama-Birmingham, United States of America

## Abstract

RhoA is a member of the Rho family small GTPases that are implicated in various cell functions including proliferation and survival. However, the physiological role of RhoA *in vivo* remains largely unknown. Here, we deleted RhoA in the B cell and hematopoietic stem cell (HSC) populations in *RhoA^flox/flox^* mice with *CD19* and *Mx* promoter-driven Cre expression, respectively. Deletion of RhoA by *CD19^Cre/+^* significantly blocked B cell development in spleen, leading to a marked reduction in the number of transitional, marginal zone, and follicular B cells. Surprisingly, neither B cell proliferation in response to either LPS or B cell receptor (BCR) engagement nor B cell survival rate *in vivo* was affected by RhoA deletion. Furthermore, *RhoA^−/−^* B cells, like control cells, were rescued from apoptosis by BCR crosslinking *in vitro*. In contrast, RhoA deficiency led to a defect in B cell activating factor (BAFF)-mediated B cell survival that was associated with a dampened expression of BAFF receptor and a loss of BAFF-mediated Akt activation. Finally, HSC deletion of RhoA by Mx-Cre severely reduced proB/preB and immature B cell populations in bone marrow while common lymphoid progenitors were increased, indicating that RhoA is also required for B cell progenitor/precursor differentiation. Taken together, our results uncover an important role for RhoA at multiple stages of B cell development.

## Introduction

B lymphocytes differentiate from bone marrow hematopoietic stem cells (HSCs) through a series of developmental stages [1]–[4]. In the first stage, B cell progenitors, or proB cells, undergo immunoglobulin heavy chain gene rearrangement to differentiate into preB cells. PreB cells then undergo immunoglobulin light chain gene rearrangement to further develop into immature B cells. Immature B cells emigrate from the bone marrow to the spleen, where they become transitional (T) B cells. Although a small proportion of T B cells mature into non- circulating marginal zone (MZ) B cells, most differentiate into follicular (FO) B cells that circulate to spleen follicles, lymph nodes, and bone marrow.

RhoA is an intracellular signal transducer of the Rho family small GTPases that cycles between an inactive GDP-bound form and an active GTP-bound form under tight regulation [5], [6]. Activated RhoA binds to a variety of effector molecules (e.g. ROCK and mDia) to modulate actin cytoskeleton organization, adhesion, migration, proliferation, and survival in mammalian cells [7]–[11]. In B cells, RhoA transduces signals from the B cell receptor (BCR) to regulate phosphatidylinositol-4, 5-bisphosphate synthesis, PLCγ2 activation, calcium influx, protein kinase D activation, and B cell proliferation [12], [13]. RhoA also plays a role in CCL19- and CCL21-induced migration of B-chronic lymphocytic leukemia cells [14]. However, the role of RhoA in B cell development remains unknown, although three closely related members of Rho GTPases, Rac1, Rac2, and Cdc42 are all required for B cell differentiation [15], [16]. The majority of published literatures for RhoA have employed overexpression of constitutively active or dominant negative RhoA mutants. While this strategy has resulted in a number of key discoveries, including the critical role of RhoA in actin stress fiber formation and cell adhesion [7], it is compromised by the promiscuous nature of these RhoA mutants [17]–[20]. For instance, RhoA dominant negative mutants sequester upstream guanine nucleotide exchange factors (GEFs) that are capable of activating additional Rho GTPases, potentially leading to non-specific phenotypes. In addition, overexpression of constitutively active GTP-bound RhoA mutants might activate a number of effectors (e.g., ROCK) shared between RhoA and other Rho GTPases, potentially causing off-target effects. Finally, a strictly balanced RhoA GTP-binding/GTP-hydrolysis cycle is required for proper signaling. As such, the overexpression of these mutants might introduce artifacts by locking RhoA into a single conformation at a fixed intracellular location. It is therefore highly desirable to use a gene targeting strategy to assess the physiological functions of RhoA.

Several studies have reported the use of *RhoA* conditional gene targeting to define RhoA functions in mice [21]–[23]. Deletion of RhoA in skin cells revealed that, while RhoA is not required for skin development, it is indispensible for the contraction and directed migration of primary keratinocytes. In the nervous system, RhoA maintains adherens junctions and modulates neuronal cell proliferation. Furthermore, contrary to the conventional view that RhoA is essential for actin cytoskeleton rearrangement and cell adhesion, RhoA-deficient primary mouse embryonic fibroblasts (MEFs) display normal actin stress fiber and focal adhesion complex formation. Nonetheless, RhoA is critical for MEF cell proliferation.

To assess the physiological role of RhoA in B cell development, we generated mouse strains deficient for RhoA expression in either B cells or HSCs by crossing *RhoA^flox/flox^* mice with *CD19^Cre/+^* or *Mx-Cre* transgenic mice. Using these targeted deletion models, we demonstrate that RhoA is crucial for B cell development and for B cell activating factor (BAFF)-mediated B cell survival, but not for BCR-mediated proliferation and survival.

## Results

### Generation of RhoA-deficient B cells by *CD19^Cre/+^*

**Figure 1:**
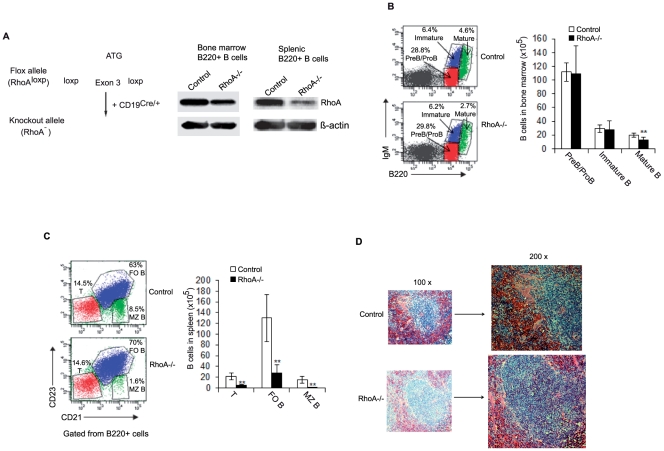
B cell-specific deletion of RhoA impairs splenic B cell development. (A) Generation of *RhoA^−/−^* B cells. Left, the loxP/Cre-mediated gene targeting strategy to generate the *RhoA* knockout allele (*RhoA^−^*) in B cells. Right, Western blot showing RhoA expression in B220^+^ B cells purified from bone marrow and spleen of *CD19^Cre/+^; RhoA^+/+^* (control) and *CD19^Cre/+^; RhoA^flox/flox^* (*RhoA^−/−^*) mice. (B) Bone marrow cells from control and *RhoA^−/−^* mice were stained with antibodies against B220 and IgM and analyzed by flow cytometry (left). The number of B cell subsets was calculated by multiplying the total number of bone marrow cells by the percentage of each subset of cells (right). n = 5. (C) Splenocytes from control and *RhoA^−/−^* mice were stained with antibodies against B220, CD21 and CD23 and analyzed by flow cytometry (left). The number of B cell subsets was calculated by multiplying the total number of splenocytes by the percentage of each subset of cells (right). T: transitional B cells, FO B: follicular B cells, and MZ B: marginal zone B cells. n = 5. (D) Spleen sections from control and *RhoA^−/−^* mice, stained with hematoxylin and eosin. Data are representative of 3 mice. Error bars represent mean ± SD. **p<0.01. Statistical analysis was performed using a Student's unpaired t-test with a two-tailed distribution.

To study the role of RhoA in B cell development, *RhoA^flox/flox^* mice [22], [23] were crossbred with *CD19^Cre/+^* transgenic mice to generate *CD19^Cre/+^; RhoA^flox/flox^* (*RhoA^−/−^*) mice (Fig. 1A). Because *CD19^Cre/+^* transgenic mice are generated by inserting the *Cre* gene into the *CD19* locus, the transgenic mice are depleted of one copy of the *CD19* gene, which may affect B cell development [24]. We thus used *CD19^Cre/+^; RhoA^+/+^* mice, but not *RhoA^flox/flox^* mice, as control for *RhoA^−/−^* mice. B220^+^ B cells were purified from the bone marrow and spleen of *RhoA^−/−^* and control mice. Western blot analysis indicated that RhoA expression was dramatically reduced in splenic B cells, but less so in bone marrow B cells in the mutant mice (Fig. 1A).

### Effects of RhoA deletion on B cell development

To determine if RhoA deficiency affects B cell development, we performed FACS analysis of B cells at various stages of differentiation. We did not detect significant changes in either the percentage or number of ProB/PreB cells (B220^lo^IgM^−^) or immature B cells (B220^lo^IgM^+^) in *RhoA^−/−^* mice (Fig. 1B), likely due to the demonstrated inefficient RhoA deletion in bone marrow B cells (Fig. 1A). In contrast, *RhoA^−/−^* mice displayed a reduction in recirculating B cells (B220^hi^IgM^+^) in bone marrow (Fig. 1B), suggesting that late splenic B cell development is altered by the disruption of RhoA expression. Indeed, the number of all splenic B subsets, including T (B220^+^CD21^−^CD23^−^), MZ (B220^+^CD21^+^CD23^−^) and FO (B220^+^CD21^+/−^CD23^+^) B cells, was drastically decreased in *RhoA^−/−^* mice, although the frequency of T and FO B cells was only marginally affected (Fig. 1C). In agreement, hematoxylin and eosin staining of *RhoA^−/−^* spleen sections revealed lymphoid hypoplasia and loss of follicular architecture (Fig. 1D). Moreover, B cells were decreased in the lymph nodes and blood in the absence of RhoA (data not shown). Taken together, these results suggest that RhoA is critical for the late B cell development.

### Effects of RhoA deletion on B cell proliferation and survival

**Figure 2:**
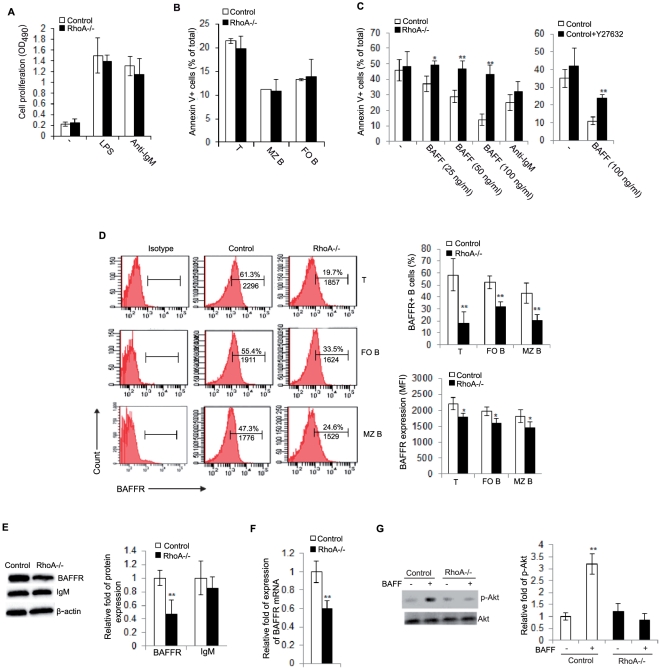
RhoA is necessary for BAFF-mediated B cell survival but not proliferation. (A) Splenic B220^+^ B cells from *CD19^Cre/+^; RhoA^+/+^* (control) and *CD19^Cre/+^; RhoA^flox/flox^* (*RhoA^−/−^*) mice were cultured for 48 hours on 96-well plates (4×10^5^ cells/well) with or without (−) 2 µg/mL anti-IgM F(ab')_2_ antibody or LPS. Cell growth rate was analyzed using the CellTiter 96® AQ_ueous_ Non-Radioactive Cell Proliferation Assay (MTS) kit. Data are expressed as absorbance OD_490_. n = 5. (B) Splenocytes from control and *RhoA^−/−^* mice were stained with anti-B220, -CD21, and -CD23 antibodies followed by Annexin V staining. The cells were then analyzed by flow cytometry. T: transitional B cells, FO B: follicular B cells, and MZ B: marginal zone B cells. n = 5. (C) Splenic B220^+^ B cells from control and *RhoA^−/−^* mice were cultured for 72 hours on 96-well plates (2×10^5^ cells/well) with or without (−) 2 µg/mL anti-IgM F(ab')_2_ antibody or indicated concentrations of BAFF (left). Alternatively, control B cells were incubated with or without (−) BAFF and/or Y27632 (10 µM) (right). The cells were then stained with Annexin V and analyzed by flow cytometry. n = 5. (D) Splenocytes from control and *RhoA^−/−^* mice were stained with antibodies against B220, CD21, CD23 and BAFFR, and then analyzed by flow cytometry. The numbers above bracketed lines indicate the percentage of BAFFR^+^ cells in each B cell subset and the numbers below the bracketed lines indicate mean fluorescence intensity (MFI) of BAFFR in each B cell subset (left). The percentage of BAFFR^+^ cells and MFI of BAFFR were averaged from 5 mice for each genotype (right). (E) Splenic B220^+^ B cells from control and *RhoA^−/−^* mice were subjected to Western blot for BAFFR and IgM (left). β-actin serves as loading control (left). The protein expression was quantified and normalized to β-actin and the data are expressed as fold of expression (right). n = 3. (F) Splenic B220^+^ B cells from control and *RhoA^−/−^* mice were analyzed for BAFFR mRNA levels by quantitative RT-PCR. The expression of GAPDH was used to normalize samples and the relative fold of expression is shown. n = 4. (G) Splenic B220^+^ B cells from control and *RhoA^−/−^* mice were stimulated with or without BAFF(100 ng/mL) for 30 min and then subjected to Western blot (left). Phospho (p)-Akt (S473) is quantified and normalized to total Akt and the data are expressed as relative fold of p-Akt (right). n = 3. Error bars represent mean ± SD. **p<0.01. *p<0.05. Statistical analysis was performed using a Student's unpaired t-test with a two-tailed distribution.

Cell proliferation is crucial for B cell development [4]. To explore the mechanisms underlying impaired splenic B cell development in RhoA-deficient mice, we determined the proliferative capacity of *RhoA^−/−^* splenic B cells upon *in vitro* culture with either LPS or anti-IgM F(ab')_2_ antibody to crosslink BCR. Surprisingly, we found that under either condition the *RhoA^−/−^* B cell proliferation profile did not statistically differ from control cells (Fig. 2A). These results suggest that RhoA is dispensable for Toll-like receptor- or BCR-mediated B cell proliferation. Along with recent studies showing that RhoA-deficient neural progenitor cells are hyperproliferative while RhoA-deficient MEFs have impaired proliferation [22], [23], it appears that RhoA plays cell type-specific roles in the regulation of cell proliferation.

B cell survival constitutes another important requirement for B cell development [2]. Since RhoA reportedly regulates cell survival [6], we examined the survival index of RhoA-deficient B cells by Annexin V staining and found that *Ex vivo* splenic B cells from *RhoA^−/−^* mice had no detectable survival defect (Fig. 2B). Moreover, control and *RhoA−/−* B cells cultured *in vitro* with anti-IgM F(ab')_2_ antibody exhibited a similar increase in survival induced by BCR ligation (Fig. 2C). However, *RhoA^−/−^* B cells were resistant to B cell survival factor BAFF-mediated apoptosis inhibition while control B cells were rescued from apoptosis by BAFF in a dose-dependent manner (Fig. 2C). Interestingly, treatment with Y27632, a chemical inhibitor of the RhoA downstream effector ROCK, partially blunted the survival response of control B cells to BAFF (Fig. 2C), suggesting that RhoA modulates BAFF-mediated B cell survival, at least in part, through activation of ROCK. The lack of a survival response of *RhoA^−/−^* B cells to BAFF correlates with a reduction of BAFF receptor (BAFFR) on T, FO, and MZ B cells, as demonstrated by a reduction of BAFFR^+^ cells and the mean fluorescence intensity per cell (Fig. 2D). The reduction of cell surface BAFFR is not likely due to a trafficking defect since Western blot revealed that *RhoA^−/−^* B cells had a 50% reduction in BAFFR protein expression compared to control B cells (Fig. 2E). Quantitative real-time PCR analysis demonstrated a reduction of BAFFR mRNA in *RhoA^−/−^* B cells, suggesting that the reduction in BAFFR protein is due to reduced transcription and not a reduction in protein stability (Fig. 2F). Indeed, RhoA deficiency completely abolished BAFF-mediated Akt activation, likely due to decreased BAFFR expression (Fig. 2G). In contrast to BAFFR, RhoA deficiency had no effect on BCR expression as revealed by comparable levels of IgM in control and *RhoA^−/−^* B cells (Fig. 2E), in agreement with the normal proliferative or survival response of *RhoA^−/−^* B cells to BCR ligation. Collectively, these results suggest that RhoA selectively regulates BAFF-mediated B cell survival by controlling BAFFR expression.

### Effects of HSC deletion of RhoA on B cell precursor development in bone marrow

**Figure 3:**
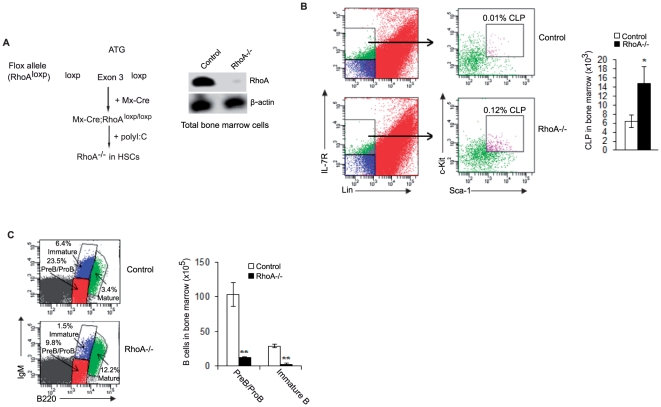
RhoA is required for bone marrow B cell development. (A) *Mx-Cre; RhoA^flox/flox^(RhoA^−/−^)* mice were injected with polyI:C to delete RhoA in HSCs (left). *RhoA^flox/flox^* mice were used as control and also subjected to polyI:C injection. Whole bone marrow was isolated from *RhoA^−/−^* and control mice and analyzed for RhoA expression by Western blot (right). (B) Bone marrow cells from *RhoA^−/−^* and control mice were stained for IL-7R, lineage (Lin) markers (B220, CD3, CD4, CD8, Gr1, CD11b and TER119), Sca-1, and c-Kit followed by flow cytometry analysis. Common lymphoid progenitor (CLP) cells (Sca1^+^c-kit^+^) were gated from a Lin^−^IL-7R^+^ cell population (left). The number of CLPs was calculated by multiplying the total number of bone marrow cells by the percentage of CLP cells (right). n = 4. (C) Bone marrow cells from *RhoA^−/−^* and control mice were stained with antibodies against B220 and IgM and analyzed by flow cytometry (left). The number of B cell subsets was calculated by multiplying the total number of bone marrow cells by the percentage of each subset of cells (right). n = 4. Error bars represent mean ± SD. **p<0.01. *p<0.05. Statistical analysis was performed using a Student's unpaired t-test with a two-tailed distribution.

As previously demonstrated, *CD19^Cre/+^*-mediated RhoA deletion in bone marrow B cells is inefficient (Fig. 1A), precluding further examination of the role of RhoA in their development. Instead, we employed an alternatively method to generate *RhoA^−/−^* HSCs by crossing *RhoA^flox/flox^* with *Mx-Cre* transgenic mice and inducing Cre expression in *Mx-Cre; RhoA^flox/flox^* mice with polyI:C (Fig. 3A). *RhoA^flox/flox^* littermates were used as control and also treated with polyI:C. Since *Mx-Cre; RhoA^flox/flox^* mice die within eight days after polyI:C induction, we analyzed the mice at day six and found that RhoA was almost completely absent from the bone marrow (Fig. 3A). FACS analysis indicated that both the percentage and number of common lymphoid progenitors (CLP) were elevated in *Mx-Cre; RhoA^flox/flox^* mice (Fig. 3B), but the proB/preB and immature B cell subsets were markedly decreased (Fig. 3C). The percentage, but not number, of recirculating mature B cells in bone marrow was increased in *Mx-Cre; RhoA^flox/flox^* mice (Fig. 3C and data not shown), likely due to a compensatory effect of the decreased proB/preB and immature B cells. These results suggest that RhoA deficiency inhibits bone marrow B cell development at the CLP differentiation stage. Thus, RhoA also plays a critical role in early B cell development.

## Discussion

In this study, we have shown that RhoA deletion in B cells by *CD19^Cre/+^* causes a defect in splenic B cell development that is associated with an impaired BAFF-mediated survival response. Accordingly, BAFFR expression and BAFFR-mediated Akt activation are dampened in RhoA-deficient B cells. We have also demonstrated that depletion of RhoA from HSCs by Mx-Cre results in an earlier developmental defect at the transition of CLP to proB/preB cells. These data suggest that RhoA plays critical roles in both early and late stages of B-cell development.

The B cell developmental phenotypes in *RhoA^−/−^* mice show some similarities as well as differences from mice deficient for the other Rho family GTPases, Cdc42, Rac1 and/or Rac2 [15], [16], [25]. For instance, *RhoA^−/−^*, *Cdc42^−/−^* and *Rac1^−/−^Rac2^−/−^* mice all have decreased numbers of T, FO and MZ B cells while *Rac2^−/−^* mice show a reduction only in MZ B cells [15], [16], [25]. Additionally, defective BAFF-mediated B cell survival signaling is observed in all *RhoA^−/−^*, *Cdc42^−/−^* and *Rac1^−/−^Rac2^−/−^* mice [15], [16]. In contrast, RhoA deficiency has no effect on BCR-mediated cell proliferation, whereas ablation of either Cdc42, Rac2 or both Rac1 and Rac2 dampens BCR signaling and cell proliferation [15], [16], [25]. Moreover, deletion of Rac2 or both Rac1 and Rac2, but not Cdc42, compromises B cell migration [16], [25], [26], which is thought to play a role in B cell development [27]. It remains to be determined whether depletion of RhoA affects B cell migration. These data combined with our findings suggest that different aspects of B cell development may require distinct Rho GTPases.

The lack of response of *RhoA^−/−^* B cells to BCR-mediated cell proliferation might be due to functional redundancy of other Rho GTPase family members, including Cdc42, Rac1, and Rac2, or compensatory activation of RhoB and/or RhoC [15], [16], [25]. We could not detect an effect of RhoA deficiency on bone marrow B cell development, potentially due to the poor deletion efficiency of *CD19^Cre/+^* in these cells. Indeed, *CD19^Cre/+^* is reportedly less efficient in deleting polβ in preB cells than in splenic B cells [28]. Furthermore, we have observed that *CD19^Cre/+^* only partially deletes Cdc42 in proB/preB cells [16]. As such we speculate that the residual RhoA expression in *RhoA^−/−^* bone marrow B cells is attributable to the incomplete deletion of RhoA in proB/preB cells. So to study the role of RhoA in bone marrow B cell development, we instead used Mx-Cre to remove RhoA from HSCs. Noting that *Mx-Cre; RhoA^flox/flox^* mice die within eight days after polyI:C injection, likely due to severe anemia, we analyzed bone marrow B cell differentiation at day six and found that RhoA deficiency blocked CLP differentiation to proB/preB and subsequent immature B cells. We also examined B cell maturation in the spleen of *Mx-Cre; RhoA^flox/flox^* mice, but no abnormalities were observed in the numbers of T, FO and MZ B cells (data not shown). We postulate that the intact splenic B cell differentiation in *Mx-Cre; RhoA^flox/flox^* mice may be due to a perturbation of preexisting splenic B cells that can live relatively longer than bone marrow B cells and therefore may not be lost by six days post polyI:C induction. Thus, the combined use of *Mx-Cre; RhoA^flox/flox^* and *CD19^Cre/+^; RhoA^f lox/f lox^* mouse models has allowed us to investigate specific roles for RhoA in both early bone marrow and late splenic B cell development.

The survival of peripheral B cells is regulated by BCR and BAFFR [29]. Interestingly, we find that RhoA selectively regulates BAFF/BAFFR- but not BCR-mediated survival. Hence, RhoA appears to play a stimulus-specific role in B cell survival. This concept is supported by a number of observations in other cell types. For instance, RhoA is important for thrombin-induced ICAM-1 expression, but not for the induction of ICAM-1 expression by TNFα, in endothelial cells [30]. In chondrocytes, RhoA regulates TGF-β- but not IL-1-induced actin cytoskeleton reorganization [31]. Furthermore, RhoA mediates TNFα- but not UV-induced NF-κB activation in COS-7 cells [32].

Since BAFFR expression is reduced in *RhoA^−/−^* splenic B cells, RhoA may regulate BAFF/BAFFR-mediated B cell survival by modulating BAFFR expression. In addition to its primary functions in regulating BAFF-induced B cell survival, homeostasis and differentiation [29], [33]–[36], BAFFR has also been shown to regulate the production of naturally occurring IgM, IgG1 and T cell-dependent IgG and to be involved in T-cell independent class switch to IgG and IgE [35], [36]. It is therefore plausible that RhoA might play a role in antibody formation. BAFFR has recently been shown to regulate B cell chemotaxis [37]. It is conceivable then that RhoA contributes to B cell chemotaxis via regulation of BAFFR expression. Finally, literature indicates that BAFFR is required for CD21 and CD23 expression [36], so RhoA could regulate the differentiation of FO and MZ B cells by affecting BAFFR-mediated expression of FO and MZ B cell surface markers.

## Materials and Methods

### Ethics statement

This study involved the use of mice and was carried out in strict accordance with the recommendations in the Guide for the Care and Use of Laboratory Animals of the Cincinnati Children's Hospital Research Foundation. The protocol was approved by the Committee on the Ethics of Animal Experiments of the Cincinnati Children's Hospital Research Foundation (permit Number: 8D06052). Mice were anesthetized using ketamine (80–100 mg/kg IM), aceptromazine (4–6 mg/kg IM) and atropine (0.1 mg/kg IM) and anesthesia was maintained using ketamine (30 mg/kg IM) as needed. During the course of experiments, mice were isolated in microisolator cages and cared for by trained technician and veterinarians of the Laboratory Animal Resource Center. Animals were checked daily by qualified laboratory personnel. Euthanasia was performed by CO_2_. This method was approved by the Animal Care and Use Committee of the Cincinnati Children's Hospital Research Foundation and consistent with the recommendations of the Panel on Euthanasia of the American Veterinary medical Association.

### Mouse gene targeting

Conditionally targeted *RhoA^flox/flox^* mice were generated as previously described [22], [23]. The floxed allele contains *loxP* sites flanking exon 3 of the *RhoA* allele (allele symbol: Rhoa^tm1Yuyo^ [22]). To delete RhoA *in vivo* in the B cell lineage, *RhoA^flox/flox^* mice were mated with mice expressing Cre recombinase under the control of a *CD19* proximal promoter (allele symbol: CD19^tm1(cre)Cgn^ [24]) (Jackson Laboratory). Mice used for experiments ranged in age from six to twelve weeks. To delete RhoA *in vivo* in HSCs, *Mx-Cre;RhoA^flox/flox^* mice were generated by breeding *RhoA^flox/flox^* mice to *Mx-Cre* transgenic mice carrying a bacteriophage Cre recombinase driven by an interferon-α-inducible *Mx* promoter (allele symbol: Tg(Mx1-cre)29-4Her [38]) (gift of Dr. Stuart H. Orkin, Dana-Farber Cancer institute, Boston). The expression of Mx-Cre was induced by two intraperitoneal (IP) injections of 300 µg of polyI:C (Amersham Pharmacia Biotech Inc) into six to twelve week old mice. Bone marrow cells were analyzed six days post polyI:C injection. Animals were housed under specific pathogen-free conditions in the animal facility at Cincinnati Children's Hospital Research Foundation.

### Immunoblotting

B cells were purified from bone marrow and spleen using B220 Microbeads (Miltenyi Biotec). Total bone marrow cells and purified B cells were lysed and protein content was normalized by Bradford assay. Lysates were separated by 10% sodium dodecyl sulfate polyacrylamide gel electrophoresis. Western blot was performed using the following antibodies: RhoA (67B9), phospho-Akt (D9E), and Akt (C67E7) (Cell Signaling Technology), IgM (μ chain specific) (Jackson ImmunoResearch Laboratories), and BAFFR (G-20) (Santa Cruz).

### Characterization of B cell development by cell surface staining

Single cell suspensions were prepared from bone marrow and spleen. Cells were incubated with various combinations of the following antibodies at room temperature for 20 minutes: B220 (RA3-6B2), CD21 (7G6), CD23 (B3B4), CD3 (145-2C11), CD4 (RM4-5), CD8 (53-6.7), Gr1 (RB6-8C5), CD11b (M1/70), TER119 (TER-119), c-kit (2B8), and Sca1 (D7) (BD Biosciences) and IL7Rα (A7R34) and BAFFR (eBio7H22-E16) (eBioscience). Flow cytometry analysis was performed on a FACSCanto system using FACSDiVa software (BD Biosciences).

### Cell apoptosis analysis

B cells were purified from spleen using B220 microbeads. The cells were cultured for 72 hours on 96-well plates at a density of 10^6^ cells/mL in 200 µL of B cell culture medium (10% fetal calf serum; 10 mM Hepes pH 7.3; L-glutamine; Penicillin/Streptomycin; non-essential amino acids; sodium pyruvate; and 5×10^−5^ M 2-mercaptoethanol, in RPMI-1640) in the presence or absence of 2 µg/mL of anti-IgM F(ab')_2_ antibody (Jackson Immunoresearch Laboratories), or various concentrations of recombinant human BAFF (PeproTech). The cultured B cells and *ex vivo* B cells were then stained with Annexin V (BD Biosciences) followed by flow cytometry analysis.

### Cell proliferation assay

Purified splenic B cells were cultured for 48 hours on 96-well plates at 2×10^6^ cells/mL in 200 µL of B cell culture medium in the presence or absence of 2 µg/mL of either anti-IgM F(ab')_2_ antibody or LPS. Cell growth rate was analyzed by using the CellTiter 96® AQ_ueous_ Non-Radioactive Cell Proliferation Assay (MTS) kit (Promega).

### Hematoxylin and eosin staining

Spleens were fixed in 10% buffered formalin, embedded in paraffin, sectioned at 5 µM and stained with hematoxylin and eosin.

### Gene expression analysis

RNA was isolated from splenic B cells using RNeasy Micro Kit (QIAGEN) and converted to cDNA using a High Capacity cDNA Reverse Transcription Kit (Applied Biosystems Inc). Real-time polymerase chain reaction (PCR) was performed with a Taqman system on a 7900HT Real-Time machine (Applied Biosystems Inc). The primer sequences for amplification of BAFFR cDNA were CCTCCGCTCAAAGAAGATGCA (forward primer) and GTGGAGCCCAGTTCTGT (reverse primer). Data were analyzed using SDS 2.3 software (Applied Biosystems Inc) and normalized to glyceraldehyde-3-phosphate dehydrogenase.

### Statistical Analysis

Data are expressed as the mean ± standard deviation (SD). Data were analyzed by a Student's unpaired t-test with a two-tailed distribution. Significance was accepted at p<0.05.
